# Single-cell transcriptome sequencing reveals potential novel combination of biomarkers for antibody-based cancer therapeutics in hepatocellular carcinoma

**DOI:** 10.3389/fgene.2022.928256

**Published:** 2022-09-14

**Authors:** Hong Tang, Jun Yuan, Yuan-Feng Gong, Cheng-Yang Zhang, Ming Liu, Su-Xia Luo

**Affiliations:** ^1^ Department of Internal Medicine, Affiliated Cancer Hospital of Zhengzhou University, Henan Cancer Hospital, Zhengzhou, China; ^2^ Affiliated Cancer Hospital and Institute of Guangzhou Medical University, Guangzhou, China; ^3^ School of Basic Medical Science, Guangzhou Medical University, Guangzhou, China

**Keywords:** cancer immune-therapeutics, hepatocellular carcinoma, single-cell RNA sequencing, plasma membrane protein, antibody drug, tumor heterogeneity, precision medicine, therapeutic resistance

## Abstract

**Background:** Antibody-based cancer therapeutics is developing rapidly in recent years for its advantages in precisely targeting the tumor cells. However, tumor-specific cell surface antigens are still lacking, and the heterogeneity of tumor mass greatly impeded the development of effective drugs.

**Methods:** In the present study, single-cell RNA sequencing was used to dissect tumor heterogeneity in human hepatocellular carcinoma (HCC). Tissues from different spatial regions including the tumor, para-tumor, and distant normal liver tissues were dissociated into single cells, and the gene expressions were compared in a different subpopulation of cells from these regions and validated in independent cohorts.

**Results:** A total of 28 cell clusters with different distribution patterns and gene expression profiles were identified within a heterogenous tumor and its paired liver tissues. Differentially expressed genes encoding the plasma membrane in cells with hepatic lineage were further extracted from single-cell transcriptome sequencing and validated in TCGA database. A 3-gene signature was identified to be significantly upregulated in dominant HCC tumor cell subpopulations with prognostic significance and validated in multiple independent patient cohorts.

**Conclusion:** The composition of the three plasma membrane proteins on the surface of HCC tumor cells within a heterogenous tumor might indicate poor prognostic tumor subpopulations during cancer evolution and potential therapeutic targets.

## Introduction

Hepatocellular carcinoma (HCC) is one of the most common human malignancies with poor prognosis, which ranked as the fourth cause of cancer death worldwide (El-Serag, 2011). Although surgical resection and liver transplantation have already been used for early-stage HCC treatment, however, most of the patients were diagnosed with no chance for curative therapy. The current targeted therapeutic drugs approved by the US Food and Drug Administration (FDA) for unresectable advanced HCC are multi-kinase inhibitors including sorafenib, regorafenib, and lenvatinib. However, the benefit of the patients from the therapy is very limited, with a prolonged median overall survival time of less than 3 months (Llovet et al., 2008; Bruix et al., 2017; Kudo et al., 2018).

Antibody-based cancer therapeutics is developing rapidly in recent years for its ability to precisely target the tumor cells with low side effects. There are multiple moieties of antibody-based therapeutic drugs, including the monoclonal antibody, bi-specific antibody, and antibody–drug conjugates (ADCs). The common feature of these drugs is that they can specifically recognize tumor cell surface antigens and bind to them based on the antibody variant chain. Then, tumor cells will be destroyed by either antibody-dependent cell-mediated cytotoxicity (ADCC) or the cytotoxic drugs conjugated to the antibody (Jabbour et al., 2021; Melero et al., 2021; Sangro et al., 2021). Although many new promising targets are emerging for antibody-based therapeutic drug development, the lack of suitable targets is still a major challenge, especially in solid tumors (Sano et al., 2019; Serrano et al., 2019). In addition, the tumor is highly heterogeneous, and the tumor cells may undergo clonal shift under therapeutic pressure to escape drug attack. Like other solid tumors, HCC is a dynamic disease with subpopulations of tumor cells that keep on evolving during malignant progression and therapeutic treatment. This ongoing evolution generates a diverse hierarchy of cancer cells harboring distinct molecular features and cellular identities (Tirosh et al., 2016; Lan et al., 2017; Liu et al., 2020). Although several cell surface markers for liver cancer progenitor cells have been identified in recent years, a comprehensive characterization of HCC-specific biomarkers at single-cell resolution might help identify the advantageous subclones and druggable targets for cancer treatment (Ma et al., 2007; Lee et al., 2011; Kong et al., 2021; Lee et al., 2022).

Single-cell RNA sequencing (scRNA-seq) is developing rapidly in recent years and has greatly contributed to the understanding of cancer pathogenesis at high resolution (Krieger et al., 2021; Pan and Jia, 2021; Sun et al., 2021). In contrast to bulk RNA sequencing, which cannot distinguish the proportions of distinct cellular components within the tumor mass, single-cell RNA sequencing is able to dissect the cellular compositions and characterize their molecular features in a heterogeneous tumor (Dinh et al., 2021; Dutta et al., 2022; Song et al., 2022). In addition, single-cell RNA sequencing can also monitor the transition state of tumor cells under therapeutic pressure and trace cancer cell evolution, thus facilitating the identification of novel cancer biomarkers and therapeutic targets (Kröger et al., 2019; Thong et al., 2020; Gay et al., 2021). In the present study, single-cell transcriptome sequencing was used to profile the gene expression atlas of different cellular components in HCC tumor tissues, para-tumor liver tissues, and distant normal liver tissues. The gene expression signatures were generated from cell populations of hepatic origin. In particular, genes encoding plasma membrane proteins were selected and analyzed in cell populations from different sites. A composition of the 3-gene signature was identified to be significantly upregulated in HCC tumor cells compared with their normal counterpart and as biomarkers of potential dominant subpopulations during tumor evolution. Further development of antibodies targeting those HCC tumor-specific cell surface proteins might help precisely deliver therapeutic drugs and efficiently eradicate tumor cells.

## Materials and methods

### Patient specimens

The patient’s liver tumor and adjacent tissues were obtained from a patient with clinically confirmed HCC. The patient was a male, 52 years old. Para-tumor liver tissue was collected less than 1 cm from the tumor, and distant normal liver tissue was collected more than 1 cm from the tumor. Samples were collected with the approval from the Ethics Committee of the Affiliated Cancer Hospital and Institute of Guangzhou Medical University. Informed consent was signed by the patient.

### Tissue dissociation and preparation of single-cell suspensions

The obtained tissue samples were washed two to three times in 4°C precooled 1 × PBS on ice, transferred to a sterile RNase-free culture dish, and cut into small pieces (around 0.5 mm^2^) with 10-cm surgical scissors. The tissues were washed with 1 × PBS. Non-purpose tissues such as blood stains, fatty layers, and connective tissues were removed as much as possible. The tissue samples were incubated in a constant temperature water bath or shaker at 37°C with the self-prepared hydrolysate or the hydrolysate kit (MACS Human Tumor Dissociation Kit DS_130-095-929). Incubation was terminated when the tissue mass disappeared. A 40-μm cell sieve was used to filter. The cell suspension was then centrifuged at 300 rpm at 4°C for 5 min. The supernatant was discarded, and 1 ml of resuspension buffer was added to the centrifuge tube to resuspend the cells. A measure of 3 ml of precooled erythrocyte lysis liquid was added to the tube (Solarbio), blowing and performing suction evenly. It is then incubated at 4°C for 5–10 min and centrifuged at 300 rpm at 4°C for 5 min immediately after completion. The supernatant is removed, and 1 ml of resuspension buffer (precooled 1 × PBS) was added to fully resuspend the cells. Testing was conducted with a cell counter (Countstar Rigel S2), and the concentration was adjusted according to the test result. The target concentration is 700–1200 cell/μL. The cell activity should be greater than 90%, and the agglomeration should be less than 15%. Once the final cell concentration and activity were reached, the cells were placed on ice, and the 10× genomics single-cell transcriptome chip-on-board experiment was carried out within 30 min.

### Single-cell RNA-seq library preparation and sequencing

We prepared single-cell RNA-seq libraries with Chromium Next GEM Single Cell 3′ Reagent Kits v.3.1 on the Chromium Controller (10× Genomics). We prepared single-cell suspensions from cultured cell lines, and single cells were suspended in PBS containing 0.04% BSA. The cell suspension was loaded onto the Chromium Next GEM Chip G and ran the Chromium Controller to generate single-cell gel beads in the emulsion (GEMs), according to the manufacturer’s recommendation. The captured cells were lysed, and the released RNA was barcoded through reverse transcription in individual GEMs. Barcoded, full-length cDNA was generated, and libraries were constructed, according to the performer’s protocol. The quality of libraries was assessed by Qubit 4.0 and the Agilent 2100 systems. Sequencing was performed on the Illumina NovaSeq 6000 system with a sequencing depth of at least 50,000 reads per cell and 150 bp (PE150) paired-end reads.

### Processing raw data and assays from scRNA-seq of 10× Genomics

We performed alignment to this amended reference using 10× cellranger 6.0.0, which employs the STAR sequence aligner. The reference genome was the human genome GRCh38. Overall, 12,784 cells passed the quality control. To remove the cells with low quality, cells with gene numbers over 500 and the ratio of mitochondria lower than 0.20 were maintained, and genes with at least one feature count in more than three cells were used for the following analysis. We determined the gene expression contusion unique molecular identifiers (UMIs) for each cell barcode–gene combination. Following alignment, we filtered cell barcodes to identify those which contain cells using the approach implemented in cellranger 3.0.2, and only these barcodes were considered for downstream analysis including clustering and cell-type identification and differential expression analysis by Seurat (version 4.0.1).

### Dimensionality reduction

To enable unsupervised clustering and cell-type identification, we perform dimensionality reduction with principal component analysis (PCA) on the combined set of samples for each tissue. To visualize the data, we further reduced the dimensionality of all 26,231 cells using Seurat and also used t-SNE to project the cells into 2D space. The steps include 1. using the LogNormalize method of the “Normalization” function of Seurat software to calculate the expression value of genes; 2. PCA (principal component analysis) was performed using the normalized expression value, within all the PCs, and the top 10 PCs were used to perform clustering and t-SNE analysis; 3. to find clusters, selecting the weighted shared nearest neighbor (SNN) graph-based clustering method. Marker genes for each cluster were identified by the “bimod” function (likelihood-ratio test) with default parameters *via* the FindAllMarkers function in Seurat (4.0.1). Fold Change > 1.5 and FDR < 0.1 are filtered from the calculation results of the FindAllMarkers function, and then they are sorted to the top 10 genes as the marker gene.

### Clustering and cell-type identification

We applied Louvain community detection to the nearest neighbor graph constructed in PCA space to define a cluster partition. To infer cell types, we trained a neural network classifier to predict cell ontology classes, given single-cell RNA-seq mRNA abundance profiles. First, we used SingleR (4.0.4) containing seven data sets to automate the identification of cell types. In addition to these annotations, we manually added cell state annotations to SingleR to provide a level of granularity below cell ontology classes. After completing the annotations, we further corrected the cell types using the CellMarker database (http://bio-bigdata.hrbmu.edu.cn/CellMarker/).

### Public databases and RNA-seq data sets

The gene subcellular location data were downloaded from the Human Protein Atlas (https://www.proteinatlas.org/about/download) database. TCGA RNA-seq mRNA expression data on LIHC were obtained from the LIHC project of TCGA (https://tcgadata.nci.nih.gov/tcga/) database. The data were downloaded from UCSC Xena (https://xena.ucsc.edu/). The LIHC project contains 377 primary liver cancer tissue samples and 50 normal liver tissue samples. The LIHC samples were divided into high- and low-expression groups based on a gene median expression level. Further mRNA expression data and clinical pathological data were obtained from the ICGC portal (https://dcc.icgc.org/projects/LIRI-JP). These samples belong to a Japanese population primarily infected with HBV/HCV. We used the normalized read count values given in the gene expression file. The GEO datasets GSE25097 from the Sangerbox (http://sangerbox.com/) were downloaded and analyzed. GSE25097, based on Rosetta/Merck Human RSTA Affymetrix 1.0 microarray, comprises 268 HCC tumor, 243 adjacent non-tumor, 40 cirrhotic, and 6 healthy liver samples. The GTEx datasets of bulk tissue gene expression were downloaded from the Genotype-Tissue Expression Project (GTEx: https://www.gtexportal.org/home/).

### Statistical analysis

The differential gene expression analysis among HCC cells in tumor tissue and normal hepatocytes in distal liver tissue was determined by single-cell sequencing. Genes were chosen with differential expression setting; the average of fold change (tumor/normal) ≥ 2 and *p* value ≤0.05, which encode proteins located in the cell membrane for subsequent analysis. The public databases’ differential expression profiles between tumor tissues and the normal liver tissues were generated based on the normalized expression value of RNA-seq data. Independent student’s *t*-test was used to compare the median expression level of two different groups. A one-way ANOVA test was used to compare the means between three subgroups. The test was performed in GraphPad Prism (La Jolla, CA, United States). Kaplan–Meier survival curves of the two risk groups were plotted, and the log-rank *p* value of the survival difference was calculated between them.

## Results

### Dissection of hepatocellular carcinoma patients’ tissue heterogeneity by scRNA-seq

**FIGURE 1:**
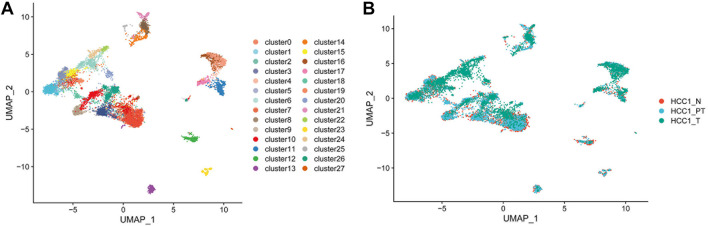
Dissection of HCC patient tissue heterogeneity by scRNA-seq. **(A)** Total of 12,784 single cells and 28 cell clusters were identified and shown with UMAP; and **(B)** distribution of cells in different samples (N, distal liver tissue; PT, proximal liver cancer tissue; T, liver cancer tissue.)

Clinically confirmed HCC tissue and paired para-tumor and distant normal liver tissues were collected from a 52-year-old male patient and dissociated into single cells for high-throughput sequencing. A total of 12,784 cells from HCC patients with liver cancer (4,285 cells), as well as from proximal (4,586 cells) and distal normal tissue (3,913 cells), were obtained. In total, 28 cell clusters were identified and shown with Uniform Manifold Approximation and Projection (UMAP) (Figure 1A). The distribution of cells in different samples is shown in Figure 1B.

### Annotation of different cell types in hepatocellular carcinoma tissues

**FIGURE 2:**
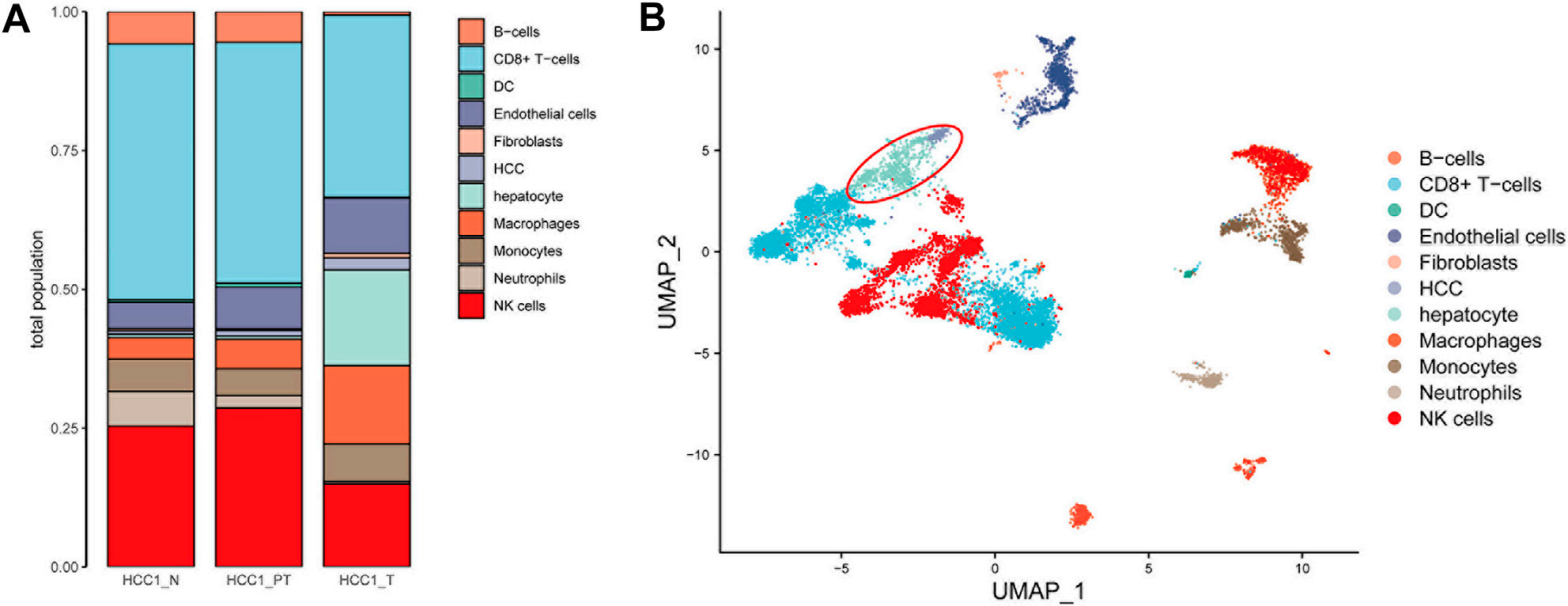
Annotation of different cell types in HCC tissues. **(A)** Number of 11 cell types in HCC tissues was calculated and shown with a bar plot; and **(B)** distribution of the cell type in the UMAP plot (liver cells are in the red circle, including hepatocytes and hepatocellular carcinoma cells).

SingleR (4.0.4) containing seven datasets was used for automatic identification of cell types. In addition to these annotations, we manually added cell-state annotations to SingleR to provide a level of granularity below cell ontology classes. As shown in Figure 2A, types of cells were identified, and most of them are immune cells, such as B cells, CD8^+^ T cells, dendritic cells, macrophages, monocytes, neutrophils, and natural killer cells (Supplementary Table S1). This finding indicated the existence of intensive immune infiltration in the tumor and para-tumor regions. In addition, fibroblasts and endothelial cells were also found in the tumor microenvironment. This indicated that the tumor mass is composed of multiple cell types with high heterogeneity. These cells may interact with hepatic cells and form a niche for tumor development. In particular, cells with hepatic origin including normal hepatocytes and HCC tumor cells were highlighted for further analysis (Figure 2B).

### Selection of tumor-specific genes encoding plasma membrane proteins from hepatic subpopulations

**FIGURE 3:**
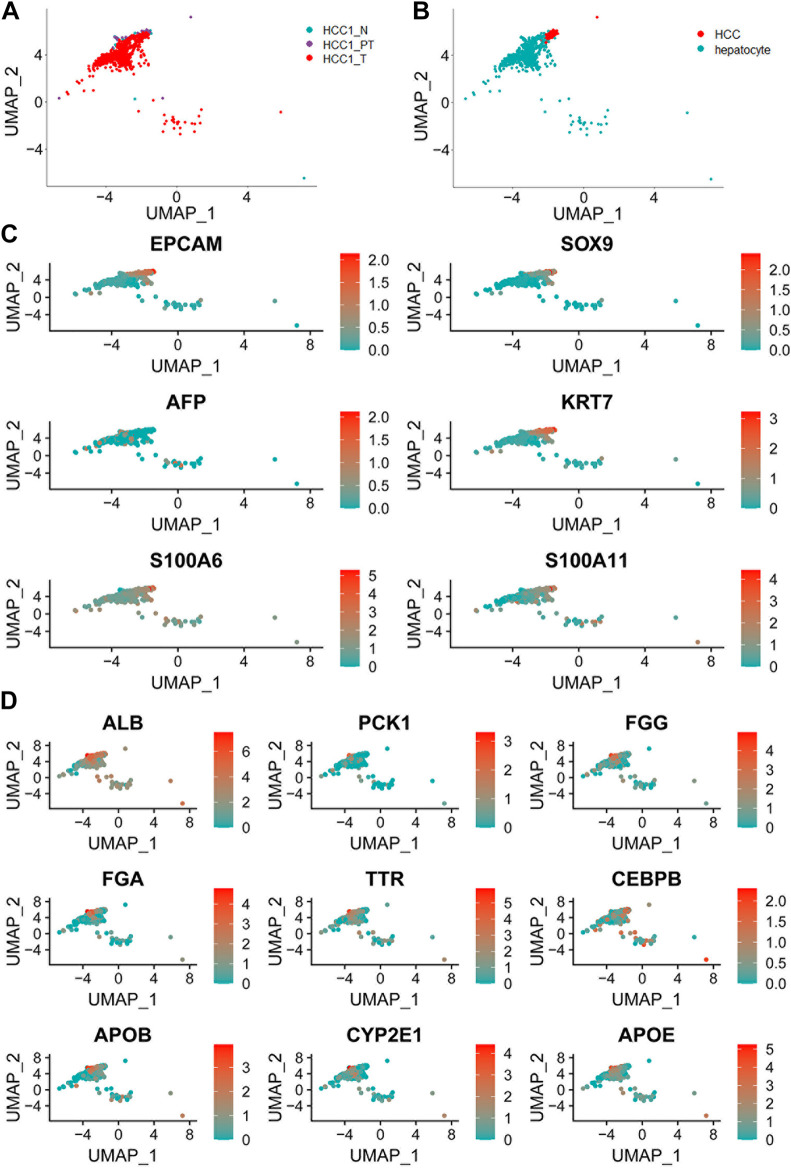
Distribution of hepatic cells in different tissue samples. **(A)** UMAP plot showing a total of 952 liver cells in different tissue samples; **(B)** UMAP plot of all hepatocytes including normal hepatocytes and hepatocellular carcinoma cells; **(C)** UMAP plot of liver cancer cell markers based on known lineage-specific marker genes (*EPCAM*, *SOX9*, *AFP*, *KRT7*, *S100A6*, and *S100A11*); and **(D)** UMAP plot of normal liver cell marker genes (*ALB*, *PCK1*, *FGG*, *FGA*, *TTR*, *CEBPB*, *APOB*, *CYP2E1*, and *APOE*).

Cell markers for normal hepatocytes and hepatocellular carcinoma cells, as reported in the current available studies, as well as in the CellMarker database (http://biobigdata.hrbmu.edu.cn/CellMarker/), were used to extract cell populations with hepatic origins from the total cells. The UMAP plots showed the distribution of hepatic cells in different tissue samples (Figure 3A). The annotated hepatocytes and HCC cells are shown in Figure 3B. The relative expression and distribution of HCC tumor-specific marker genes (*EPCAM*, *SOX9*, *AFP*, *KRT7*, *S100A6*, and *S100A11*) and hepatic lineage marker genes (*ALB*, *PCK1*, *FGG*, *FGA*, *TTR*, *CEBPB*, *APOB*, *CYP2E1*, and *APOE*) are also shown in the UMAP plots (Figure 3C and Figure 3D). To identify the potential tumor-specific markers, gene expression was compared between normal hepatocytes within the distant normal liver tissue and tumor cells within the tumor tissue. A total of 769 genes were identified by differential expression analysis with Seurat (version 4.0.1).

### Identification of a 3-gene signature significantly upregulated in hepatocellular carcinoma tumor cell subpopulations

**FIGURE 4:**
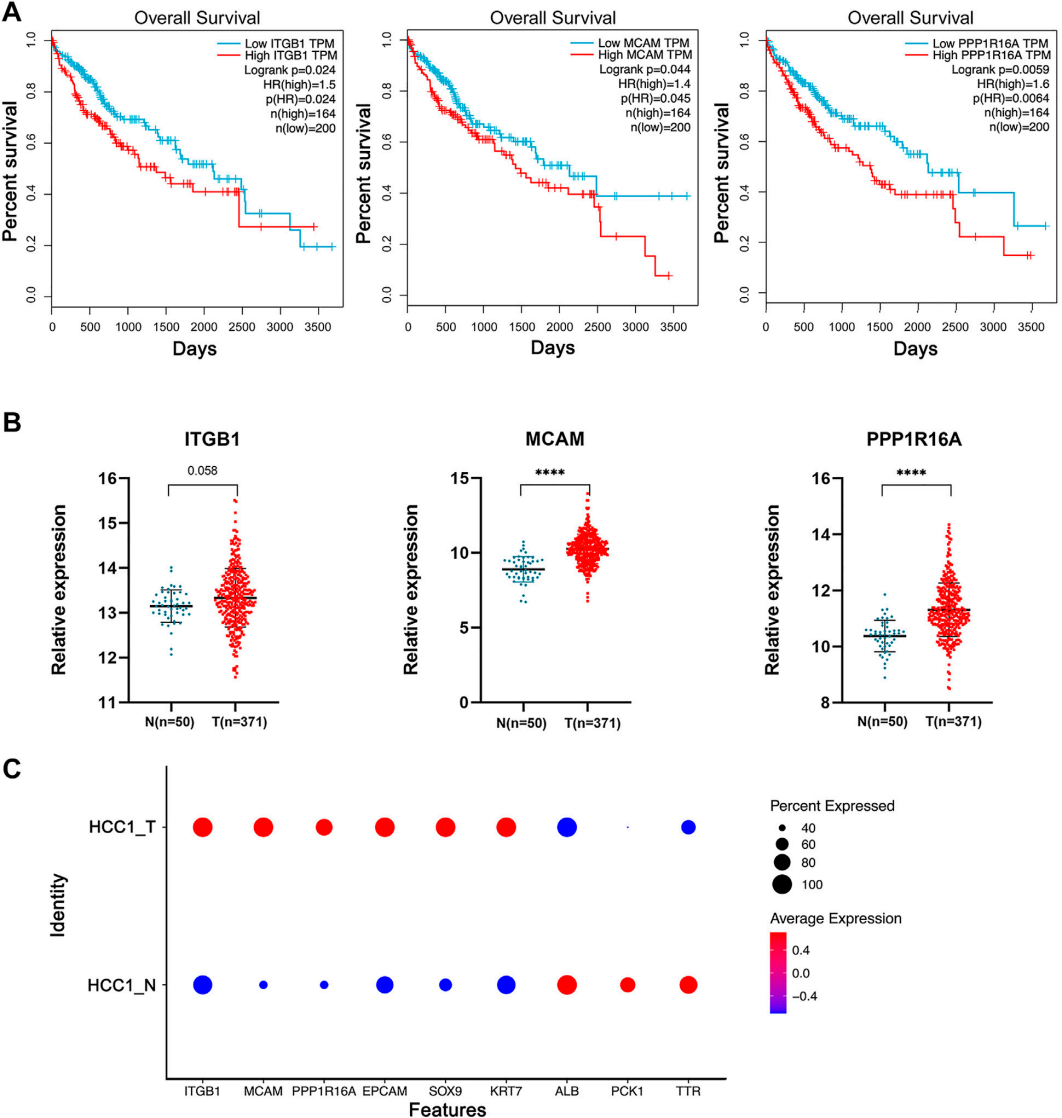
Identification of a 3-gene signature significantly upregulated in HCC tumor cell subpopulations. **(A)** TCGA-LIHC samples were divided into high- and low-expression groups based on a gene median expression level. Kaplan–Meier survival curves of the two groups were plotted, and the log-rank *p* value of the survival difference was calculated. In total, 55% was used as the cutoff value for patient subgrouping. **(B)** TCGA database differential expression profiles between tumor tissues and the normal liver tissues were generated based on the normalized expression value of RNA-seq data. Independent student’s *t*-test, ****, *p* <0.0001. **(C)** Average expression and the percentage expression of the selected signature genes in whole-cell populations.

**FIGURE 5:**
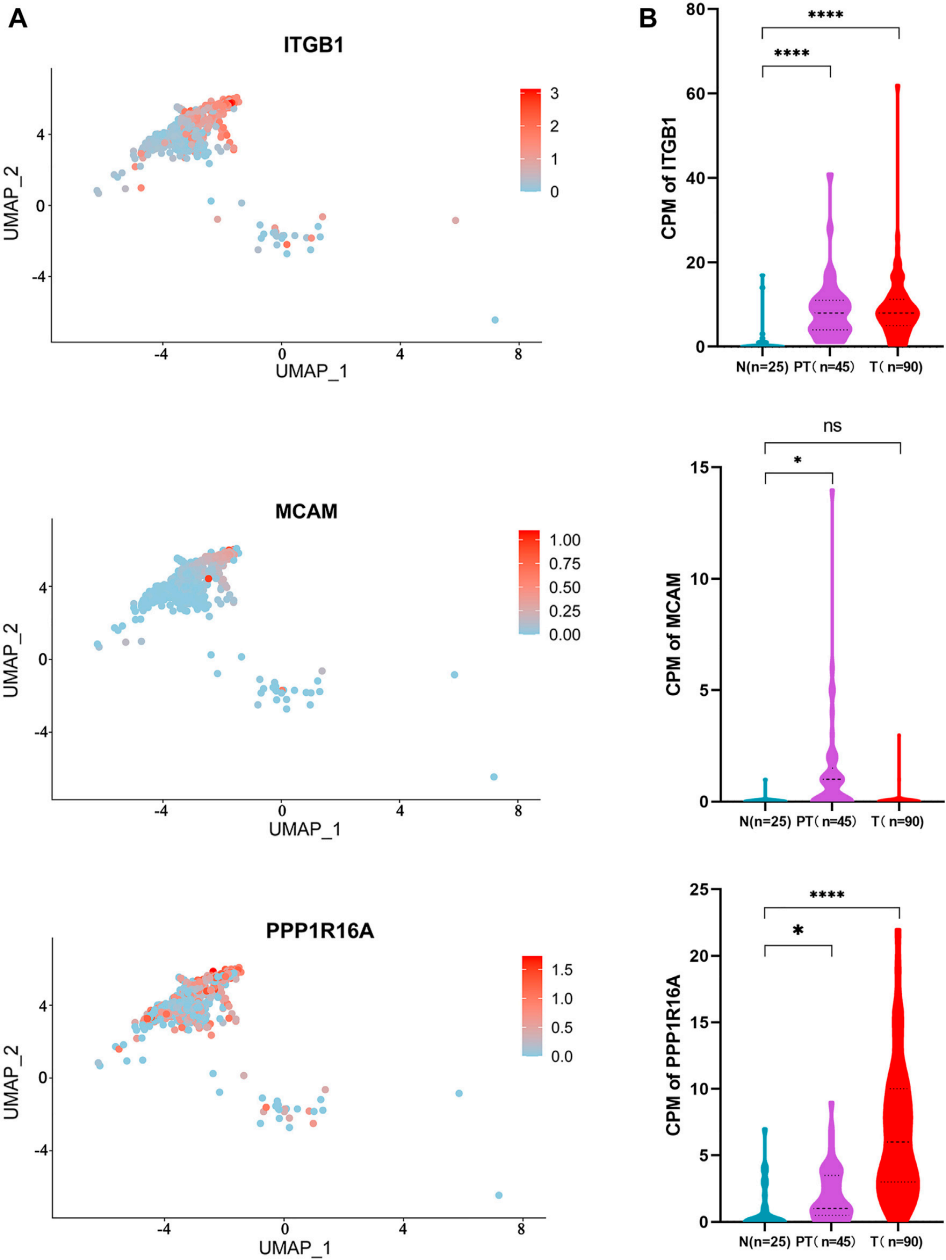
Distribution and expression of the 3-gene signature in HCC tissues. **(A)** UMAP plot showing the distribution of the three genes in HCC tissues; and **(B)** expression of the three genes in different spatial parts of the HCC tissues are shown in the violin plot. Independent student’s *t*-test, ****, *p* <0.0001.

By matching these genes to a list of genes encoding plasma membrane proteins obtained from the Human Protein Atlas (HPA) website, 110 genes were selected. A total of 42 genes with differential expression settings of average fold change (tumor/normal) ≥ 2 and *p* value ≤ 0.05 were retained for further analysis. The relative expression and clinical significance of 42 genes selected were investigated in TCGA database. The LIHC dataset from TCGA database with differential expression profiles between tumor tissues and normal liver tissues were generated based on the normalized expression value of RNA-seq data. Three genes including integrin subunit Beta 1 (ITGB1), melanoma cell adhesion molecule (MCAM), and protein phosphatase 1 regulatory subunit 16A (PPP1R16A) highly expressed in HCC and negatively correlated with patient survival were further identified (Figure 4A and Figure 4B). To dissect the gene expression pattern at single-cell resolution, both the average expression and the percentage expression of the selected signature genes were further analyzed in whole-cell populations. The average expression reflects the total gene expression level, while the percentage expression reflects the percentage of positive cells in the selected cell populations. As shown in Figure 4C, most of the signature genes were not only upregulated in individual cells but also expanded to a higher percentage of the whole-cell population in the tumor tissue. Conversely, the hepatic terminal differentiation markers were downregulated and have restricted expression only in a small percentage of cells in the tumor. These findings further confirmed that tumor cells harboring the signature genes are dominant clones during cancer evolution. The distribution of the three genes is shown in the UMAP plots (Figure 5A). The expression of the three genes was found to be upregulated both in tumor tissues and para-tumor liver tissues, compared with the distant normal liver tissues (Figure 5B).

### Validation of the 3-gene signature in independent cohorts and its potential as biomarkers during hepatocellular carcinoma evolution

**FIGURE 6:**
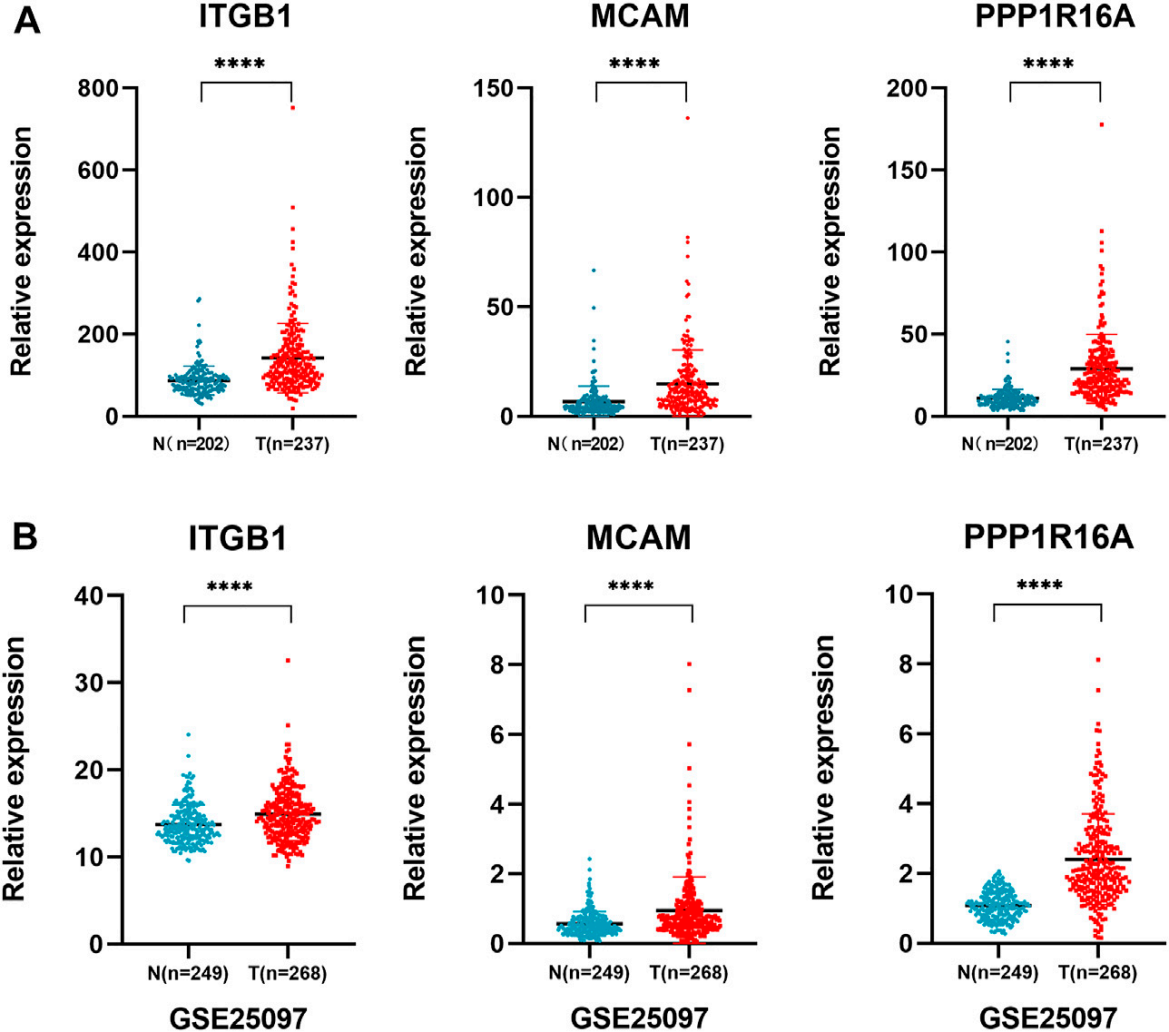
Validation of the 3-gene signature in independent cohorts. **(A)** LIRI-JP dataset was used to validate the expression of *ITGB1*, *MCAM*, and *PPP1R16A* in non-tumoral and tumoral liver tissue samples. Independent student’s *t*-test, ****, *p* <0.0001; **(B)** validation of the three gene expressions in an independent cohort GSE25097 dataset.

To further validate the findings, the relative expression of the 3-gene signature was investigated in two other independent HCC cohorts including the LIRI-JP datasets and GEO datasets (GSE25097). The results showed that the three genes were all significantly upregulated in the two cohorts of HCC patients (Figures 6A,B). These findings further confirmed that the three genes encoding cell membrane proteins are consistently upregulated in HCC tumor cells. We noticed that the absolute expression of the three genes in distant normal liver tissues from our scRNA-seq data was very low. We further checked the expression of the three genes in normal tissues or organs from the Genotype-Tissue Expression (GTEx) database. The results showed that the expression of the three genes remains at a low level in most normal tissues (Supplementary Figure S1). Further design of antibodies or antibody-conjugated drugs recognizing the 3-gene signature might help accurately target and eliminate the tumor cells.

## Discussion

The antibody-based therapeutics has greatly improved and extended to a broad range of applications in the clinic in recent years, especially in the field of cancer treatment (Weiner, 2015). Multiple strategies have been developed for antibody-based cancer therapeutics, including directly targeting the tumor cells, inhibiting tumor angiogenic endothelial cells, and blocking the immune checkpoint of T cells (Ferrara et al., 2004; Pardoll, 2012; Scott et al., 2012). In recent years, novel antibody-based cancer therapeutic moieties have emerged and gained great success in the clinic, such as antibodies conjugated with cytotoxic drugs, bispecific antibodies redirecting CD3^+^ T cells to the tumor, and chimeric antigen receptor (CAR) T cells (Zhukovsky et al., 2016; Newick et al., 2017; Drago et al., 2021). The attractive antigens for antibody-based therapeutics are the consistent expression of target molecules on malignant cells but a limited expression on benign cells. However, the tumor cells are very cunning as they can evolve under therapeutic pressure and generate antigen-negative variants. The heterogeneity of tumor mass greatly impeded the development of antibody-based therapeutic drugs. Thus, identifying the novel combination of tumor cell-specific antigens covering most subpopulations of tumor cells is greatly needed.

In this study, single-cell RNA sequencing was used to profile the cell distribution and gene expression in paired HCC tumor tissues, para-tumor liver tissues, and distant normal liver tissues. A total of 11 types of cells were identified to compose the heterogeneous HCC tumor. In particular, we extracted the gene expression data from a subpopulation of cells of hepatic origin at different spatial regions. Combining the expression and prognostic data from TCGA database, three genes encoding plasma membrane proteins covering almost all subpopulations of the tumor cells were identified to be constantly upregulated in HCC patients from multiple independent cohorts. The relative expression of the three genes was also examined in the GTEx database, which represents a panel of normal tissues. In contrast to tumor tissues, the three genes remain low or have no expression in most vital organs. These further supported their potential as tumor antigens for antibody-based therapy. Interestingly, among the three genes identified, we found that integrin Beta-1 (ITGB1) was reported to be significantly upregulated in many types of tumors, particularly those highly expressed on cancer stem cells (Gerger et al., 2011; Barnawi et al., 2019). These indicated that targeting ITGB1 might eliminate the origin of tumor cells with cancer stem cell properties. We noticed that the expression of ITGB1 did not reach statistical significance in TCGA cohort. Considering the tumor heterogeneity of the patients and the association of ITGB1 with cancer stemness properties, certain tumor subpopulations might not have significant high expression of ITGB1. However, the high abundance of ITGB1 in certain subpopulations of HCC patients might also be able to guide precision oncology further in the clinic. Taken together, we identified the composition of 3-gene signature encoding cell plasma membrane proteins significantly upregulated in HCC but remained low in most vital organs. Furthermore, targeting these tumor antigens with antibodies might facilitate the development of next-generation cancer therapeutics for HCC treatment.

## Data Availability

The original contributions presented in the study are included in the article/Supplementary Materials, further inquiries can be directed to the corresponding authors.
