# Supplementary material for: Single-cell transcriptome sequencing reveals potential novel combination of biomarkers for antibody-based cancer therapeutics in hepatocellular carcinoma
Source: Front Genet. 2022 Sep 14;13:928256. doi: 10.3389/fgene.2022.928256 (PMC9515615; doi:10.3389/fgene.2022.928256)
Supplement: Supplementary file 1 [file Table1.DOCX]

| **Cell Type** | **Clusters** | **HCC1_N** | **HCC1_PT** | **HCC1_T** | **Total** |
| --- | --- | --- | --- | --- | --- |
| B-cells | 13, 23, 27 | 227 | 252 | 25 | 504 |
| CD8+ T-cells | 0, 1, 5, 7, 15, 18, 19 | 1802 | 1991 | 1406 | 5199 |
| DC | 26 | 19 | 31 | 6 | 56 |
| Endothelial cells | 8, 14, 21 | 185 | 344 | 427 | 956 |
| Fibroblasts | 25 | 15 | 13 | 38 | 66 |
| HCC | 22 | 24 | 45 | 90 | 159 |
| Hepatocyte | 6, 24 | 25 | 28 | 740 | 793 |
| Macrophages | 4, 16 | 151 | 245 | 603 | 999 |
| Monocytes | 11, 17 | 226 | 220 | 290 | 736 |
| Neutrophils | 12 | 246 | 103 | 19 | 368 |
| NK cells | 2, 3, 9, 10, 20 | 993 | 1314 | 641 | 2948 |
| Total |  | 3913 | 4586 | 4285 | 12784 |

**Supplementary Table 1.** Cell cluster distribution in liver tissues. (N: distal liver tissue; PT: proximal liver cancer tissue; T: liver cancer tissue.)
