# Supplementary figures and images for: Single-cell transcriptome sequencing reveals potential novel combination of biomarkers for antibody-based cancer therapeutics in hepatocellular carcinoma
Source: Front Genet. 2022 Sep 14;13:928256. doi: 10.3389/fgene.2022.928256 (PMC9515615; doi:10.3389/fgene.2022.928256)

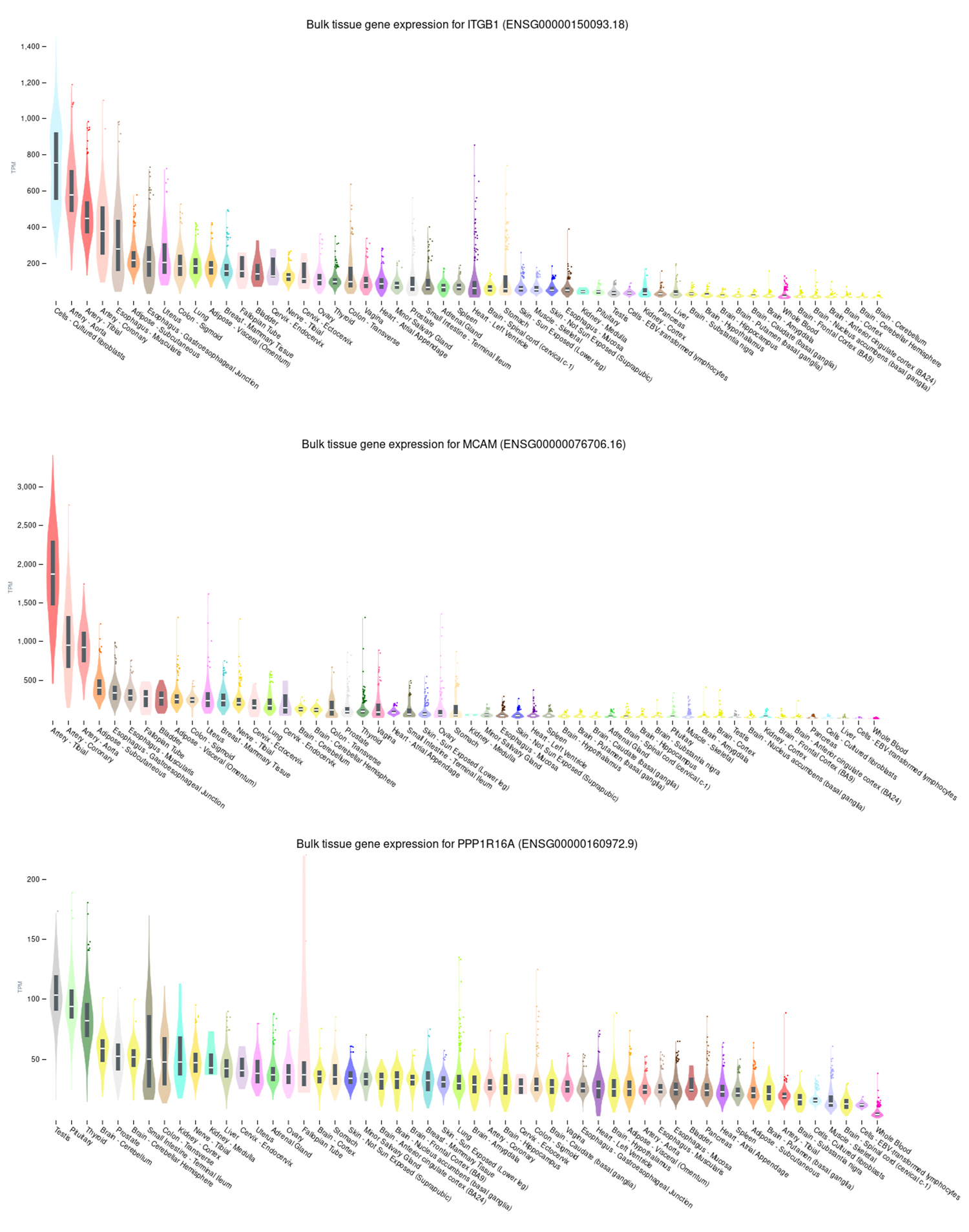

Supplement: Supplementary file 2 [file Image1.JPEG]
